# Cuneus and fusiform cortices thickness is reduced in trigeminal neuralgia

**DOI:** 10.1186/1129-2377-15-17

**Published:** 2014-03-24

**Authors:** Maud Parise, Tadeu Takao Almodovar Kubo, Thomas Martin Doring, Gustavo Tukamoto, Maurice Vincent, Emerson Leandro Gasparetto

**Affiliations:** 1 Department of Radiology, Clementino Fraga Filho University Hospital, Universidade Federal do Rio de Janeiro, Rua Rodolpho Paulo Rocco, 255, Cidade Universitária, Rio de Janeiro, CEP:21941-913, Brazil; 2 Department of Surgical Specialties, Division of Neurosurgery Pedro Ernesto University Hospital, Universidade do Estado do Rio de Janeiro, Boulevard Vinte e Oito de Setembro, 77 Vila Isabel, Rio de Janeiro, RJ CEP: 20551-900, Brazil; 3 Department of Neurology, Clementino Fraga Filho University Hospital, Universidade Federal do Rio de Janeiro, Rua Rodolpho Paulo Rocco, 255, Cidade Universitária, Rio de Janeiro, CEP:21941-913, Brazil

**Keywords:** Trigeminal neuralgia, MRI, Brain, Cortical thickness analysis, White matter, Diffusion tensor imaging

## Abstract

**Background:**

Chronic pain disorders are presumed to induce changes in brain grey and white matters. Few studies have focused CNS alterations in trigeminal neuralgia (TN).

**Methods:**

The aim of this study was to explore changes in white matter microstructure in TN subjects using diffusion tensor images (DTI) with tract-based spatial statistics (TBSS); and cortical thickness changes with surface based morphometry. Twenty-four patients with classical TN (37-67 y-o) and 24 healthy controls, matched for age and sex, were included in the study.

**Results:**

Comparing patients with controls, no diffusivity abnormalities of brain white matter were detected. However, a significant reduction in cortical thickness was observed at the left cuneus and left fusiform cortex in the patients group. The thickness of the fusiform cortex correlated negatively with the carbamazepine dose (p = 0.023).

**Conclusions:**

Since the cuneus and the fusiform gyrus have been related to the multisensory integration area and cognitive processing, as well as the retrieval of shock perception conveyed by Aδ fibers, our results support the role of these areas in TN pathogenesis. Whether such changes occurs as an epiphenomenon secondary to daily stimulation or represent a structural predisposition to TN in the light of peripheral vascular compression is a matter of future studies.

## Background

Classical trigeminal neuralgia (TN) is characterized by severe paroxysms of electric, shock-like pain limited to one or more divisions of the trigeminal nerve [1]. It is commonly evoked by trivial stimuli but frequently occurs spontaneously.

Although TN’s pathogenesis is not fully understood, there is increasing evidence suggesting that it may be related to chronic vascular compression of the trigeminal nerve root entry zone (REZ). This chronic compression may lead to focal demyelination, consequent generation of aberrant activities (ectopic discharges) and pathological cross-activation between large and small afferent fibers (ephaptic cross-talk) [2]. This peripheral process may induce secondary central changes resulting in sensitization of nociceptive neurons at the trigeminal nucleus and/or higher brain structures [3,4].

Abnormal high-frequency stimulation due to chronic pain could likewise induce morphological changes in nociception modulatory areas. Grey matter abnormalities have been observed in several chronic painful conditions like phantom pain, chronic back pain, fibromyalgia and chronic head pain [5-7]. On the other hand, white matter diffusional changes were reported in brain areas involved in sensory, modulatory and cognitive functions in temporomandibular disorder (TMD), cluster headache, migraine, complex regional pain syndrome CRPS and fibromyalgia [8-12].

Two previous publications reported on reduction of grey matter volume (GMV) in TN using voxel-based morphometry (VBM) in brain areas related to pain processing and integration [13,14]. The question remains as whether these results reflect plastic changes of the brain as a consequence of chronic nociceptive input or a causal factor for the development of TN.

The aim of this study was to investigate grey (GM) and white matter (WM) structural changes in TN and verify whether such changes would relate to disease duration, medication or sensory deficits. A MRI surface-based analysis was performed to approach GM abnormalities. For assessment of WM architectural diffusion tensor imaging (DTI) with factional anisotropy (FA) measurements and tract-based-spatial-statistics (TBSS) were used.

## Methods

### Subjects

Twenty-four consecutive patients with classical TN (18 women, 6 men) according to the International Classification of Headache Disorders version III criteria [1] were prospectively evaluated between May 2009 and May 2012 at Pedro Ernesto University Hospital. The mean age was 55.8 ± 8.5 years. Patients with symptomatic TN, other headache disorders, chronic pain elsewhere, diabetes mellitus, claustrophobia, psychiatric conditions, or previous TN operations were excluded. TN was located within the maxillary and/or mandibular branch of trigeminal nerve (V2-V3) in all but one patient, in which ophthalmic branch was also involved. TN affected the left and right sides in respectively 13 and 11 patients. The disease duration ranged from 1 to 17 years. Due to ethical reasons and to prevent head movements during scans, TN preventive medications were not discontinued.

Twenty-four matched healthy controls (12 women, 6 men, mean age 56.3 ± 7.8 years) were also included. Clinical neurological examination was unremarkable in the control group. This study was conducted in accordance with the Declaration of Helsinki and was approved by the ethical committee of Clementino Fraga Filho University Hospital (Study 062/09). All patients and controls gave written informed consent for the participation in the study.

### Measurement of tactile and pain perception thresholds

Sensory and pain perception thresholds were obtained by using bipolar electrical stimulation with surface electrodes placed on the forehead above the eyebrow (V1); on the cheek (V2) and on the chin (V3) just lateral to the mental foramen. Constant-current square-wave electrical stimuli were delivered at a 3 Hz stimulation rate with 200 ms pulse duration and varying intensity (Medelec stimulator - Vickers Medical- UK). The patient was asked to state when stimuli of increasing current intensity became noticeable (detection threshold) and “definitely” painful (pain threshold). Thresholds were measured in all trigeminal divisions and compared to the opposite side. Threshold values were determined by the method of limits, i.e. the average from five or more successive observations.

### Imaging parameters

All subjects underwent scanning in a 1.5 T MRI system (Avanto, Siemens Medical System, Erlangen, Germany) fitted with an 8-channel phased-array head coil, the following sequences of the brain obtained: 1) T1-weight 3D high-resolution magnetization-prepared rapid acquisitions with gradient echoes (3D-MPRAGE) sequence (128 slices; 1 × 1 × 1.3 mm^3^ voxels; 256 × 256 matrix size; field of view = 256 mm; echo time = 3.39 ms; repetition time = 2.530 ms; flip angle 7°; 1.3 mm thick slices). 2) Echo-planar diffusion-weighted images, with bipolar diffusion gradients in 30 orthogonal directions (122 × 122 matrix size, field of view = 256, repetition time = 10.1 s , echo-time = 94 ms, slices = 73, slice thickness = 2 mm, interslice gap = 0). To optimize the measurement of diffusion in the brain, only two b values were used (b0 = 0; b1 = 900 s/mm^2^).

To minimize motion artifacts the head of the subjects were fixed in the head coil. All images were investigated to be free of motion or ghosting.

### DTI analysis

For voxelwise diffusion modeling, diffusion data were analyzed using FMRIB’s Diffusion Toolbox within FSL 4.1 (http://www.fmrib.ox.ac.uk/fsl) [15]. After performing eddy current correction and brain extraction, FA images for all subjects were created by fitting a tensor model to the raw diffusion data. Voxelwise statistical analysis of the FA data was carried out using TBSS (Tract-Based Spatial Statistics) [16], part of FSL. All subjects’ FA data were aligned into a common space using the nonlinear registration tool FNIRT, which uses a b-spline representation of the registration warp field [17]. The FMRIB58_FA standard-space template was used as the target in TBSS (http://www.fmrib.ox.ac.uk/fsl/data/FMRIB58_FA.html). Next, the mean FA image was created and thinned to create a mean FA skeleton, which represents the centers of all tracts common to the group. Each subject’s aligned FA data were then projected onto this skeleton, and the resulting data were fed into voxelwise cross-subject statistics for all voxels with FA ≥ 0.30 to exclude peripheral tracts with significant inter-subject variability. The voxelwise analysis was performed using permutation-based inference (5000 permutations) corrected for multiple comparisons, with a threshold-free cluster enhancement (TFCE) and significance level of p < 0.05. Corrected TFCE p-value images were computed to enable the identification of differences in the FA areas between patients and healthy control subjects. The WM tracts were then identified using the Johns Hopkins University white matter tractography atlas and the International Consortium for Brain Mapping DTI-81 white matter labels atlas, both of which are available within FSL.

### Subcortical volumetry and cortical thickness analysis

Automatic subcortical volumetric segmentation and cortical thickness evaluation was performed using the FreeSurfer® (FS) image analysis suite (v.5.0.0 Martinos Center for Biomedical Imaging, Harvard-MIT, Boston, MA), which is documented and freely available for download (http://surfer.nmr.mgh.harvard.edu/). The technical details of these procedures were described previously [18]. Briefly, this fully automated process includes motion correction, removal of non-brain tissue, automated Talairach transformation, segmentation of the subcortical white matter and deep grey-matter volumetric structures (including hippocampus, amygdale, caudate, putamen, and ventricles), intensity normalization, and cortical reconstruction. This segmentation procedure assigns a neuroanatomical label to every voxel in the MR image volume. The method is based on probabilistic information estimated from a manually labeled training set. The Markov random field theory is applied, where the probability of a label at a given voxel is computed not just in terms of the grey-scale intensities and prior probabilities at that voxel, but also as a function of the labels in a neighborhood around the voxel in question. This is important for correct differentiation between the hippocampus and amygdala, which have similar grey-scale values. The results of the automatic segmentations were reviewed and any errors were corrected. Correction for the intracranial volume (ICV), including white matter, grey matter, and cerebrospinal fluid, was also estimated for each subject using FS. Individuals with a large intracranial volume tend to have larger subcortical structures; therefore, correction by the intracranial volume is crucial in volume quantification of the subcortical structures. This correction can be simply done by dividing numerically the volume of the subcortical structure through the ICV.

Cortical thickness maps were calculated for each subject. The mean cortical thickness in regions-of-interest in the groups were computed and statistically compared (p < 0.01) by a single-binary application included in the FreeSurfer distribution, Qdec (Query, design, estimate, contrast). Correction for multiple comparisons was made by Qdec using Monte-Carlo simulation (p = 0.05). Procedures for the accuracy of cortical thickness measurements have been validated with histological analysis [19] and manual measurements [20].

### Statistical analysis

Demographic characteristics, perception thresholds and subcortical volumetry data were compared using the non-parametric Mann-Whitney U-test. Student T-test was used to compare threshold values in each trigeminal division in the affected side with the unaffected side. Pearson correlation coefficients were used to evaluate whether the GM/WM changes correlated with disease duration, carbamazepine doses, sensory and pain perception thresholds. SPSS 13.0 (SPSS, Chicago IL, USA) was used for data analysys. Data shown as mean ± SD. The level of significance was set at p < 0.05.

## Results

### Patient demographics

Patients demographics are shown in Table 1. Patients and control groups did not differ in age (patients: 55.8 ± 8.5 years; controls: 56.3 ± 7.8 years; p = 0.88). All patients scored TN paroxysms as 10 in a visual analog pain scale and remained pain free between the attacks. A 200-1800 mg/day Carbamazepine (CBZ) regimen was used by all subjects except two. There was a strong positive correlation between the CBZ dose and the disease duration TN (r = 0.489, p = 0.015).

**Table 1:** Characteristics of TN patients

**Patient**	**Gender**	**Age (years)**	**TN duration (years)**	**Pain distribution**	**Side**	**CBZ dose (mg/day)**
1	M	65	2	V2	R	1200
2	F	65	5	V2 + V1	L	200
3	F	40	4	V2	R	1000
4	F	62	8	V2 + V3	L	400
5	F	65	9	V2 + V3	L	800
5	F	53	4	V2 + V3	R	200
7	F	64	10	V3	L	800
8	F	62	5	V2	L	800
9	F	52	12	V3	R	1000
10	M	54	4	V2	R	400
11	F	57	11	V2 + V3	L	1800
12	F	48	5	V2	R	0
13	F	62	10	V2	R	800
14	F	53	10	V2 + V3	R	800
15	F	53	1	V3	L	0
16	M	49	9	V2	R	1800
17	F	62	17	V2 + V3	L	800
18	M	40	5	V3	L	800
19	M	53	6	V2 + V3	R	1200
20	M	37	5	V2	L	400
21	F	56	12	V2 + V3	L	800
22	F	67	6	V2	L	600
23	F	61	1	V2	R	200
24	F	61	10	V2	L	1200

*TN* = trigeminal neuralgia; *F* = female; *M* = male; *R* = right; *L* = left; V1 = ophthalmic branch; V2 = maxillary branch; V3 = mandibular branch; *CBZ* = carbamazepine.

### Tactile and pain perception thresholds

**Figure 1:**
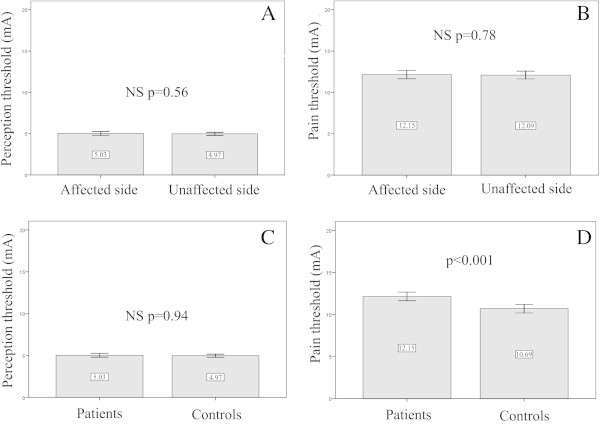
**Perception (A) and pain threshold (B) in patients with TN.** There was no difference between the affected side and the unaffected side. Differences in perception **(C)** and pain **(D)** threshold, between TN patients and controls. Patient’s pain threshold was significantly higher than controls (p < 0.001).

The tactile perception thresholds were 5.03 ± 0.9 and 4.9 ± 0.7 mA on the symptomatic and non-symptomatic sides respectively (p = 0.94). The pain perception thresholds were accordingly 12.1 ± 2.1 and 12.1 ± 1.9 mA (p = 0.78). There was no difference between tactile perception thresholds in patients (5.03 ± 0.8 mA) and controls (4.9 ± 0.7 mA) p = 0.61. However, the pain perception threshold was significantly higher bilaterally (symptomatic and non-symptomatic sides), in patients (12.3 ± 2.1 mA vs. 10.7 ± 2.1 mA, p < 0.001 - Figure 1). There was no significant correlation between the pain perception threshold and the carbamazepine dose (p = 0.11).

### White matter results

TBSS analysis revealed no significant differences in WM skeleton FA values between TN and healthy controls.

### Subcortical volumetry

Compared with controls, TN patients had significant volumetric reduction of mid-anterior portion of corpus callosum (p = 0.04). This reduction was not correlated with duration of disease or carbamazepine dose. There was no significant difference in thalamus volumetry between TN patients and controls or between the ipsilateral and contralateral thalamus in the patients group.

### Cortical thickness analysis

**Figure 2:**
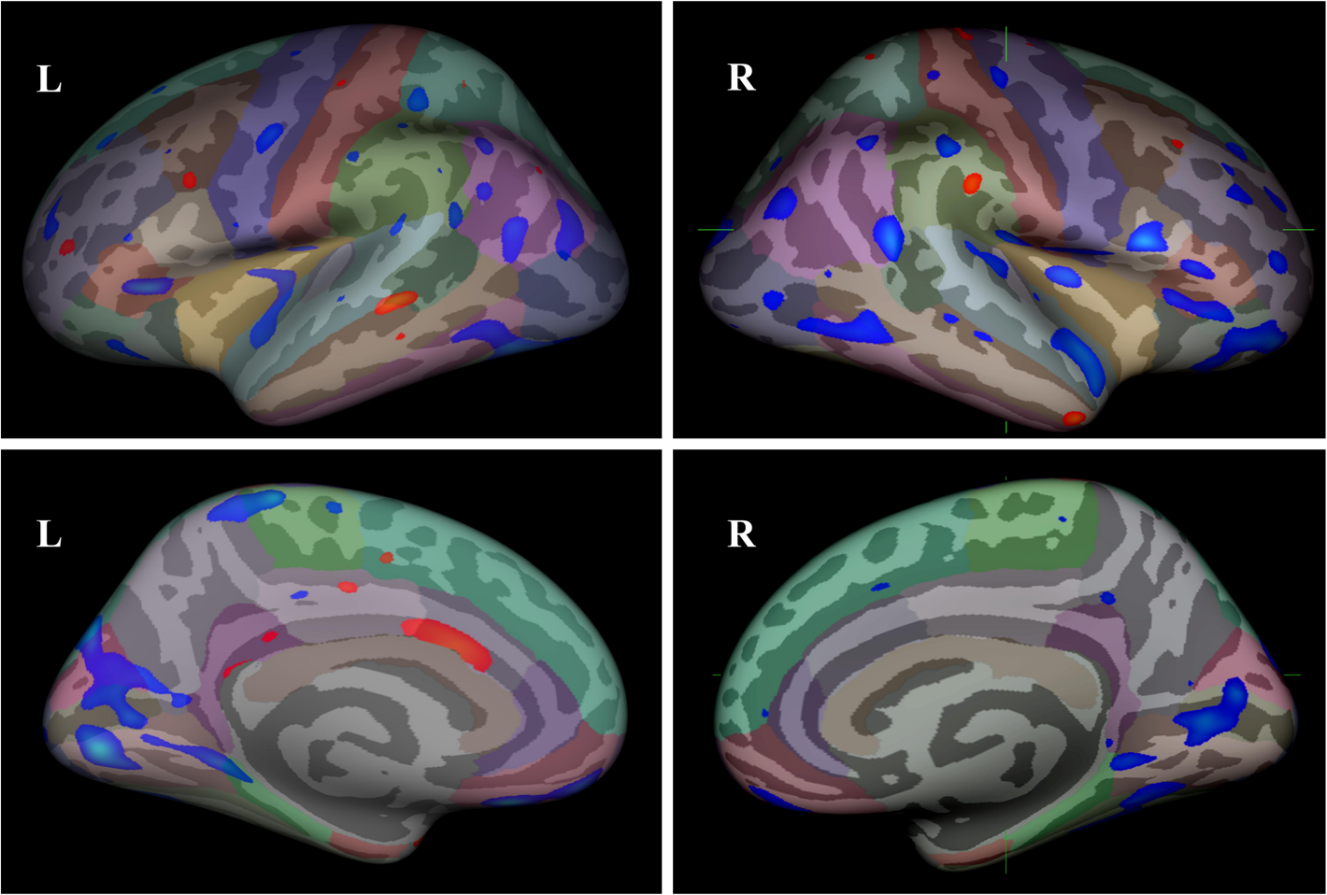
**Group differences in cortical thickness between TN patients and controls.** Blue regions indicate areas showing thickness reduction and red regions indicate thickness increase in TN patients when compared to controls at a threshold p < 0,01, uncorrected. It was observed cortical thickness changes in areas that include orbitofrontal cortex, insula, motor cortex, anterior cingulate gyrus and temporal lobe. All these regions, involved in supraspinal nociceptive processing, show grey matter alterations in different types of chronic pain.

**Figure 3:**
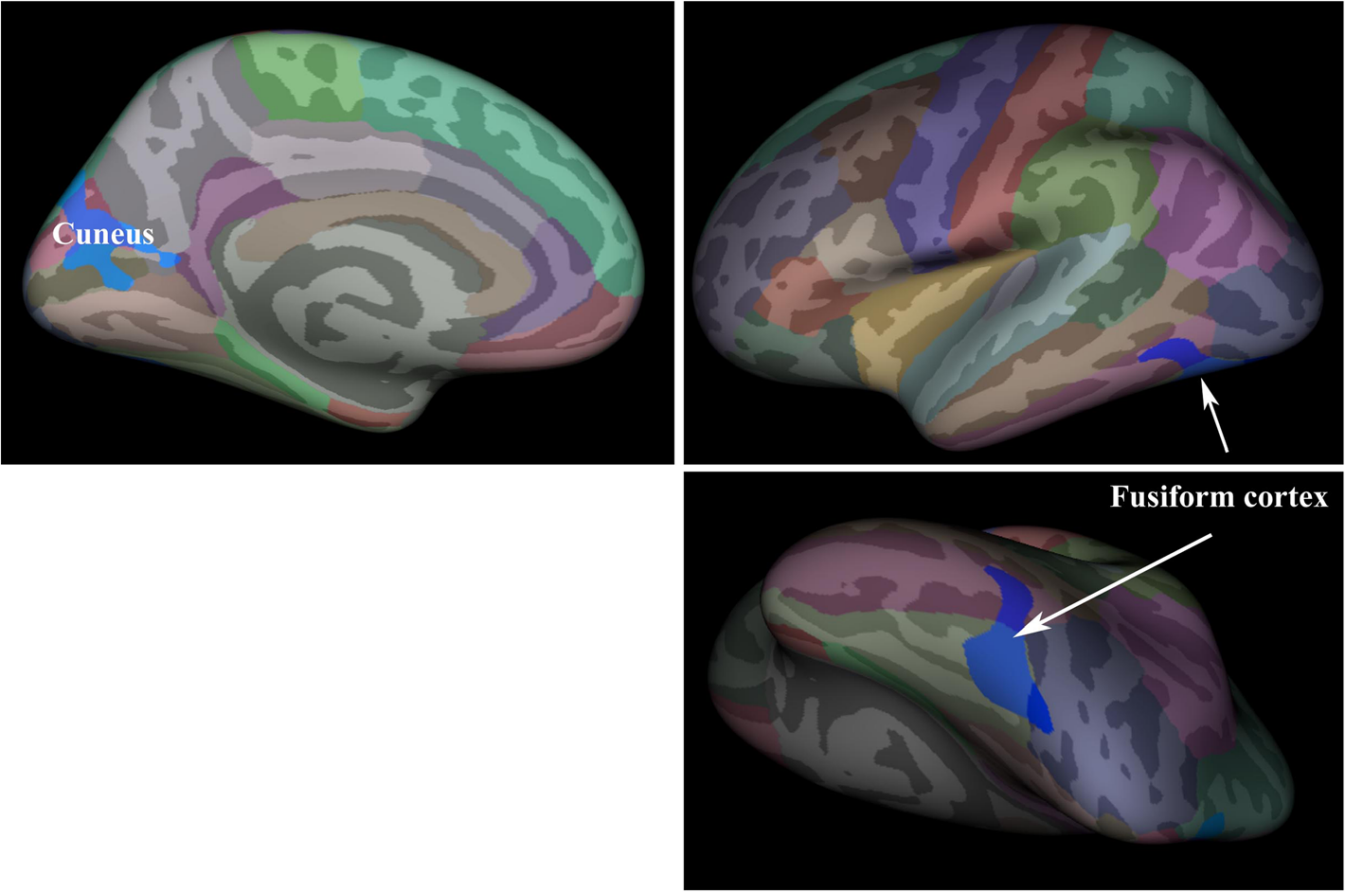
**Group differences in cortical thickness between patients and controls after correction for multiple comparisons.** The inferior temporal gyrus (fusiform cortex) and cuneus/pre-cuneus showed significant thickness reduction (blue areas) in TN patients. The thickness reduction of fusiform cortex was negatively correlated to carbamazepine doses (p = 0.023 rho 1.000 -0.463).

According to a single-binary application analisys (p < 0.01) TN patients presented increased cortical thickness in some areas including sensory, premotor, prefrontal, middle temporal cortex and cingulate gyrus; and decrease in others, such as the motor, insular, orbito-frontal, cuneus/precuneus and inferior temporal/fusiform cortices (Figure 2). After correction for multiple comparisons, only the inferior temporal/fusiform cortex and cuneus/precuneus areas remained significantly thinner (p = 0,05) (Figure 3). Cortical thickness at these areas correlated neither with disease duration nor with tactile and pain perception thresholds. However, a significant negative correlation between fusiform cortex thickness and carbamazepine dose was noticed (r = - 0.463, p = 0.023).

Four patients re-examined 6 months after successful microvascular decompression showed no significant difference between the pre- and postoperative findings.

## Discussion

Although the sensory function is roughly normal in patients with TN, subtle tactile and thermal sensory deficits may occur at the trigeminal territory as detected by sophisticated quantitative sensory testing [21]. A delayed conduction in large afferent fibres has been detected in 50% of TN patients even in the absence of any clinical hypoesthesia [22]. A conduction delay and a nociceptive laser-evoked potential amplitude reduction have been demonstrated ipsilaterally in 51% of TN subjects [23], indicating a dysfunction in Aδ and C fibres. The present study sought to detect these trifling changes - expectantly reflecting axonal lesions, demyelination and/or conduction block - as assessed by tactile and painful threshold in order to correlate them with possible WM diffusional and GM morphological changes. No significant differences in tactile and pain thresholds between the symptomatic and non-symptomatic sides or in tactile threshold between patients and controls were noticed. However, the pain threshold in patients was slightly and significantly increased (p ≤ 0.001). Bilateral activation of the descending diffuse noxious inhibitory controls (DNIC) with release of endorphins, due to the repetition of painful paroxysms of TN [24] could explain this finding, but this seems unlikely since during pain threshold determination no neuralgic pain putatively activating the inhibitory control has been triggered. A second possibility would be the antinociceptive effect of CBZ, which has been described in rats [25,26] and TN patients after noxious laser stimulation [23].

### TN and white matter abnormalities

The results of this investigation suggest that the brain white matter tracts microstructure is spared in TN, as shown by the absence of DTI abnormalities.

Subtle abnormalities reveled by FA decrease have been detected in the trigemino-thalamic and thalamocortical tracts in migraine patients [10]. Subsequent studies using TBSS analysis in other pain conditions (fibromyalgia [12], complex regional pain [11], cluster headache [9], temporomandibular disorders [8]) have reported abnormalities in white matter tracts involved in sensory, modulatory and cognitive functions.

The present results could be interpreted in two different ways. First, changes in diffusion could be too subtle or restricted to be identified by TBSS analysis. The sample size seems adequate as abnormalities have been detected in about seven cluster headache patients using the same technique [9]. Second, there are really no changes in diffusion in TN differently to migraine. Perhaps because TN is a paroxysmal pain, unlike the complex regional pain, TDM or fibromyalgia and/or does not involve in principle a genetic predisposition as in migraine. It seems that the diffusional abnormalities in the trigeminal root on the symptomatic side which would correspond to a focal demyelination and/or axonal loss [27] have no impact in the intracranial white matter tracts.

Nevertheless, we found a significant volume reduction (p = 0.04) of the mid-anterior corpus callosum that connects the motor areas of hemispheres. Clinical and experimental studies suggest an inhibitory effect of pain on the motor cortex [28-30]. Da Silva reported on reduced thickness of the primary motor cortex contralaterally to a trigeminal neuropathic pain, which could be hypothetically explained by inhibition of facial and cervical muscles to avoid pain triggering [31]. As patients with TN also limit talking, chewing and facial movements to avoid pain triggering [32,33], this could be also justify our findings.

### TN and grey matter abnormalities

We found changes in cortical thickness in TN patients as compared with controls. Cortical thickness changes, either increase or reduction, involve supraspinal nociceptive processing areas, such the orbitofrontal cortex, the insula and motor cortex, the anterior cingulate gyrus and temporal lobe, similarly to the other in pain syndromes . However, after the correction for multiple comparisons only the inferior temporal/fusiform cortex and cuneus/pre-cuneus regions remained significanty thinner (p = 0,05). In addition, the inferior temporal gyrus/fusiform cortex thickness showed a negative correlation with CBZ dose, the larger the cortical thickness, the lower the medication usage.

Pain-related grey matter changes have been reported in several pain conditions. A meta-analysis of 30 published VBM studies in 15 chronic pain conditions has shown that cortical changes (increase or decrease of grey matter) vary among different pain syndromes but overlap in areas such as cingulate and insular cortex, temporal lobe, frontal and prefrontal cortex, thalamus and basal ganglia, motor cortex, brainstem and dorsolateral prefrontal cortex, all brain areas involved in supraspinal nociceptive processing [34]. In an heterogeneous group including both TN and trigeminal neuropathy, Gustin et al. [13] found grey matter reduction in the primary somatosensory cortex, anterior insula, putamen and nucleus acumbens. The thalamic volume decrease was only seen in patients with trigeminal neuropathy but not TN. An specific study of morphometric changes in TN [14] revealed brain matter volume reduction in the primary somatosensory and orbitofrontal cortices as well as anterior cingulate, cortex insula, secondary somatosensory cortex, thalamus, putamen, caudate nucleus, dorsolateral prefrontal cortex, precuneus and cerebellum. With these results Oberman et al. [14] concluded that the GMV reduction areas were nonspecific and would reflect only the chronic pain.

Our results are similar to the previous studies in pain syndromes only in a single-binary comparison (p < 0.01), which could be explained by a lack of statistical power in this group of patients or most likely by methodological differences. Surface-based analyses provides a more precise measure of cortical thickness as VBM analysis reflects a mixed measure of grey matter, including cortical surface area or cortical folding, as well as cortical thickness [35].

After multiple comparisons only cuneus/precuneus and inferior temporal/fusiform cortex remained significantly thinner in the TN group. Although cuneus and precuneus areas are classically related to visual information processing, they are limited by the parieto-occipital sulcus, a multisensory integration area [36]. One of the cuneus function seems to be to integrate the somato-sensory information with other sensory stimuli and cognitive processes such as attention, learning and memory [37]. These areas are activated together with sensory/motor areas and structures related to pain in response to pricking sensation generated by a thermal painful stimulus in trigeminal and extra-trigeminal territory [38,39] and after selective stimulation of Aδ fibers [40]. An EEG-MEG study in neurogenic pain patients showed precuneus and cuneus hyperactivation in patients with TN but not with lower limb pain [41]. In line with the present study, Oberman et al. [14] found a reduction in GMV in the left precuneus in TN patients not related to the pain duration, attack frequency or VRS. On the contrary, cluster headache patients have an increase in the cuneus GMV [42]. Pain is very intense in both TN and cluster headache, indicating that the opposite results in this respect cannot be explained by differences in pain severity. The pain location, mostly to the lower face in TN as opposed to retro-ocular, fronto-temporal areas in cluster headache; together with the clear pathophysiological differences, central/hypothalamic cantered in cluster headache versus peripheral in TN, could explain the contrast in GMV findings among these disorders.

A significant reduction in cortical thickness was noticed in the left fusiform cortex, an area involved with body and face recognition [43]. It is also activated during postoperative pain [44] and by stimulation of Aδ fibers as well as the cuneus [40].

The perception of painful electrical shock seems activate a number of cerebral areas, namely left fusiform cortex, hippocampus, primary and associative visual cortex extending to the cuneus and precuneus as well as a more anterior network all belonging to dopaminergic pathways [45]. The activity of this areas was negatively correlated to pain intensity rating, especially at the left fusiform gyrus [45]. This posterior network, activated during electric shock stimulation, may be involved in a mental imagery process related to the retrieval of shock perception [45]. The pain in TN is shock-like in nature and could activate similar regions.

Volumetric changes of the left fusiform gyrus have been found in patients with migraine. Compared to controls, this region was significantly atrophied in migraine without aura, while in migraine with aura it was increased. In the most of patients the migraine was bilateral [46]. Pain anticipation and perception have been associated with the left fusiform gyrus, where the activity was negatively correlated with the pain rating [45]. It is possible that the left fusiforme cortex reduction may not depend on the side where the pain occurs.

The role of CBZ on the present results cannot be overlooked as the inferior temporal/fusiform cortex thickness reduction was negatively correlated with the CBZ dose, which had a positive correlation with the illness duration. Chronic CBZ was shown to change cellular signaling in rats, which could interfere with the functional and structural responses of various brain areas [47]. It remains to be determined whether CBZ affects grey matter thickness or not.

The cornerstone of TN is the electric shock-like pain evoked at trigger zones by light touch. It seems related to the ephaptic cross-talk between Aβ fibres mediating light touch and small-myelinated Aδ fibres close to the trigeminal nerve root entry zone, where there a focal demyelination is caused by neurovascular compression. Our study showed a thickness reduction of the fusiform cortex and cuneus, structures that can be activated by Aδ fibres stimulation and would be involved in a mental imagery process related to the retrieval of shock. These data strongly suggest that such cortical areas are involved in TN pathogenesis. However, our results do not elucidate whether these changes reflect plastic changes of the brain as a consequence of multiple, daily shock-like pain conveyed by Aδ fibres or a causal factor in the development of TN. Although a partial reversal of morphologic changes of the brain after pain treatment has been reported Rodriguez-Raecke [48], the four patients re-scaned 6 months after successful microvascular decompression showed no evolutive changes. It is possible that either the follow-up was too short or TN actualy requires an interplay between peripheral vascular compression and GM structural predisposition in order to develop, which is supported by the occasional presence of asymptomatic neurovascular compressions. Future studies may address this issue with longer follow-up.

## Conclusions

The present study provides evidence for GM abnormalities in areas usually involved with several chronic painful conditions, but also in specific areas as the cuneus and fusiform cortex, which may be related to retrieval of shock perception conveyed by Aδ fibers in TN. No white matter microstructure changes were identified in TN.

## Abbreviations

TN: Trigeminal neuralgia; REZ: Root entry zone; TMD: Temporomandibular disorder; CRPS: Complex regional pain syndrome; GMV: Grey matter volume; VBM: Voxel-based morphometry; GM: Grey matter; WM: White matter; MRI: Magnetic resonance image; DTI: Diffusion tensor imaging; TBSS: Tract-based-spatial-statistics; V1: Ophthalmic branch; V2: Maxillary branch; V3: Mandibular branch; MPRAGE: Magnetization-prepared rapid acquisitions with gradient echoes; FA: Factional anisotropy; TBSS: Tract-based-spatial-statistics; TFCE: Threshold-free cluster enhancement; FS: FreeSurfer; ICV: Intracranial volume; CBZ: Carbamazepine; VRS: Visual rate scale.

## Competing interests

None of the authors have received financial support for the generation of this article. The authors have no personal or institutional interest in any of the drugs, materials or devices described in this article.

## Authors’ contributions

Conception, design of the work, interpretation of data and draft the article were done by: MP, EG and MV. Acquisition and analysis of data were done by: TK, TD and GT. All the authors have read and approved the paper.

## References

[B1] ICHD-III The International classification of headache disorders: 3rd edition (beta version) Cephalalgia 2013 15 629 808 doi:10.1177/0333102413485658 23771276 10.1177/0333102413485658

[B2] Love S Coakham HB Trigeminal neuralgia: pathology and pathogenesis Brain 2001 15 2347 2360 10.1093/brain/124.12.2347 11701590

[B3] Moisset X Villain N Ducreux D Serrie A Cunin G Valade D Calvino B Bouhassira D Functional brain imaging of trigeminal neuralgia Eur J Pain 2011 15 124 131 10.1016/j.ejpain.2010.06.006 20609605

[B4] Obermann M Yoon MS Ese D Maschke M Kaube H Diener HC Katsarava Z Impaired trigeminal nociceptive processing in patients with trigeminal neuralgia Neurology 2007 15 835 841 10.1212/01.wnl.0000269670.30045.6b 17724285

[B5] Apkarian AV Sosa Y Sonty S Levy RM Harden RN Parrish TB Gitelman DR Chronic back pain is associated with decreased prefrontal and thalamic gray matter density J Neurosci 2004 15 10410 10415 10.1523/JNEUROSCI.2541-04.2004 15548656 PMC6730296

[B6] Kuchinad A Schweinhardt P Seminowicz DA Wood PB Chizh BA Bushnell MC Accelerated brain gray matter loss in fibromyalgia patients: premature aging of the brain? J Neurosci 2007 15 4004 4007 10.1523/JNEUROSCI.0098-07.2007 17428976 PMC6672521

[B7] May A Chronic pain may change the structure of the brain Pain 2008 15 7 15 10.1016/j.pain.2008.02.034 18410991

[B8] Moayedi M Weissman-Fogel I Salomons TV Crawley AP Goldberg MB Freeman BV Tenenbaum HC Davis KD White matter brain and trigeminal nerve abnormalities in temporomandibular disorder Pain 2012 15 1467 1477 10.1016/j.pain.2012.04.003 22647428

[B9] Teepker M Menzler K Belke M Heverhagen JT Voelker M Mylius V Oertel WH Rosenow F Knake S Diffusion tensor imaging in episodic cluster headache Headache 2012 15 274 282 10.1111/j.1526-4610.2011.02000.x 22082475

[B10] DaSilva AF Granziera C Tuch DS Snyder J Vincent M Hadjikhani N Interictal alterations of the trigeminal somatosensory pathway and periaqueductal gray matter in migraine Neuroreport 2007 15 301 305 10.1097/WNR.0b013e32801776bb 17435592 PMC3745625

[B11] Geha PY Baliki MN Harden RN Bauer WR Parrish TB Apkarian AV The brain in chronic CRPS pain: abnormal gray-white matter interactions in emotional and autonomic regions Neuron 2008 15 570 581 10.1016/j.neuron.2008.08.022 19038215 PMC2637446

[B12] Lutz J Jager L de Quervain D Krauseneck T Padberg F Wichnalek M Beyer A Stahl R Zirngibl B Morhard D Reiser M Schelling G White and gray matter abnormalities in the brain of patients with fibromyalgia: a diffusion-tensor and volumetric imaging study Arthritis Rheum 2008 15 3960 3969 10.1002/art.24070 19035484

[B13] Gustin SM Peck CC Wilcox SL Nash PG Murray GM Henderson LA Different pain, different brain: thalamic anatomy in neuropathic and non-neuropathic chronic pain syndromes J Neurosci 2011 15 5956 5964 10.1523/JNEUROSCI.5980-10.2011 21508220 PMC6632967

[B14] Obermann M Rodriguez-Raecke R Naegel S Holle D Mueller D Yoon MS Theysohn N Blex S Diener HC Katsarava Z Gray matter volume reduction reflects chronic pain in trigeminal neuralgia Neuroimage 2013 15 352 358 23485849 10.1016/j.neuroimage.2013.02.029

[B15] Smith SM Jenkinson M Woolrich MW Beckmann CF Behrens TE Johansen-Berg H Bannister PR De LM Drobnjak I Flitney DE Niazy RK Saunders J Vickers J Zhang Y De SN Brady JM Matthews PM Advances in functional and structural MR image analysis and implementation as FSL Neuroimage 2004 15 Suppl 1 S208 S219 15501092 10.1016/j.neuroimage.2004.07.051

[B16] Smith SM Jenkinson M Johansen-Berg H Rueckert D Nichols TE Mackay CE Watkins KE Ciccarelli O Cader MZ Matthews PM Behrens TE Tract-based spatial statistics: voxelwise analysis of multi-subject diffusion data Neuroimage 2006 15 1487 1505 10.1016/j.neuroimage.2006.02.024 16624579

[B17] Rueckert D Sonoda LI Hayes C Hill DL Leach MO Hawkes DJ Nonrigid registration using free-form deformations: application to breast MR images IEEE Trans Med Imaging 1999 15 712 721 10.1109/42.796284 10534053

[B18] Fischl B Dale AM Measuring the thickness of the human cerebral cortex from magnetic resonance images Proc Natl Acad Sci USA 2000 15 11050 11055 10.1073/pnas.200033797 10984517 PMC27146

[B19] Rosas HD Liu AK Hersch S Glessner M Ferrante RJ Salat DH Van Der KA Jenkins BG Dale AM Fischl B Regional and progressive thinning of the cortical ribbon in Huntington’s disease Neurology 2002 15 695 701 10.1212/WNL.58.5.695 11889230

[B20] Salat DH Buckner RL Snyder AZ Greve DN Desikan RS Busa E Morris JC Dale AM Fischl B Thinning of the cerebral cortex in aging Cereb Cortex 2004 15 721 730 10.1093/cercor/bhh032 15054051

[B21] Bowsher D Miles JB Haggett CE Eldridge PR Trigeminal neuralgia: a quantitative sensory perception threshold study in patients who had not undergone previous invasive procedures J Neurosurg 1997 15 190 192 10.3171/jns.1997.86.2.0190 9010417

[B22] Leandri M Parodi CI Favale E Early trigeminal evoked potentials in tumours of the base of the skull and trigeminal neuralgia Electroencephalogr Clin Neurophysiol 1988 15 114 124 10.1016/0168-5597(88)90069-X 2449329

[B23] Cruccu G Leandri M Iannetti GD Mascia A Romaniello A Truini A Galeotti F Manfredi M Small-fiber dysfunction in trigeminal neuralgia: carbamazepine effect on laser-evoked potentials Neurology 2001 15 1722 1726 10.1212/WNL.56.12.1722 11425940

[B24] Rehberg B Baars JH Kotsch J Koppe P von Dincklage F Comparison of trigeminal and spinal modulation of pain and nociception Int J Neurosci 2012 15 298 304 10.3109/00207454.2011.649868 22225522

[B25] Sahebgharani M Hossein-Abad AA Zarrindast MR On the mechanism of carbamazepine-induced antinociception in the formalin test Int J Neurosci 2006 15 1097 1113 10.1080/00207450600808669 16861171

[B26] Naseri K Sabetkasaei M Moini ZT Saghaei E Carbamazepine potentiates morphine analgesia on postoperative pain in morphine-dependent rats Eur J Pharmacol 2012 15 332 336 10.1016/j.ejphar.2011.10.026 22061686

[B27] Leal PR Roch JA Hermier M Souza MA Cristino-Filho G Sindou M Structural abnormalities of the trigeminal root revealed by diffusion tensor imaging in patients with trigeminal neuralgia caused by neurovascular compression: a prospective, double-blind, controlled study Pain 2011 15 2357 2364 10.1016/j.pain.2011.06.029 21835547

[B28] Farina S Valeriani M Rosso T Aglioti S Tamburin S Fiaschi A Tinazzi M Transient inhibition of the human motor cortex by capsaicin-induced pain. A study with transcranial magnetic stimulation Neurosci Lett 2001 15 97 101 10.1016/S0304-3940(01)02297-2 11698155

[B29] Romaniello A Cruccu G McMillan AS Arendt-Nielsen L Svensson P Effect of experimental pain from trigeminal muscle and skin on motor cortex excitability in humans Brain Res 2000 15 120 127 10.1016/S0006-8993(00)02856-0 11056191

[B30] Svensson P Miles TS McKay D Ridding MC Suppression of motor evoked potentials in a hand muscle following prolonged painful stimulation Eur J Pain 2003 15 55 62 10.1016/S1090-3801(02)00050-2 12527318

[B31] DaSilva AF Becerra L Pendse G Chizh B Tully S Borsook D Colocalized structural and functional changes in the cortex of patients with trigeminal neuropathic pain PLoS One 2008 15 10 e3396 10.1371/journal.pone.0003396 18923647 PMC2561059

[B32] Cheshire WP Trigeminal neuralgia feigns the terrorist Cephalalgia 2003 15 230 10.1046/j.1468-2982.2003.00484.x 12662192

[B33] Tolle T Dukes E Sadosky A Patient burden of trigeminal neuralgia: results from a cross-sectional survey of health state impairment and treatment patterns in six European countries Pain Pract 2006 15 153 160 10.1111/j.1533-2500.2006.00079.x 17147591

[B34] May A Structural brain imaging: a window into chronic pain Neuroscientist 2011 15 209 220 10.1177/1073858410396220 21489967

[B35] Hutton C Draganski B Ashburner J Weiskopf N A comparison between voxel-based cortical thickness and voxel-based morphometry in normal aging Neuroimage 2009 15 371 380 10.1016/j.neuroimage.2009.06.043 19559801 PMC2741580

[B36] Calvert GA Crossmodal processing in the human brain: insights from functional neuroimaging studies Cereb Cortex 2001 15 1110 1123 10.1093/cercor/11.12.1110 11709482

[B37] Price DD Psychological and neural mechanisms of the affective dimension of pain Science 2000 15 1769 1772 10.1126/science.288.5472.1769 10846154

[B38] de Leeuw R Davis CE Albuquerque R Carlson CR Andersen AH Brain activity during stimulation of the trigeminal nerve with noxious heat Oral Surg Oral Med Oral Pathol Oral Radiol Endod 2006 15 750 757 10.1016/j.tripleo.2005.12.018 17138177

[B39] Fulbright RK Troche CJ Skudlarski P Gore JC Wexler BE Functional MR imaging of regional brain activation associated with the affective experience of pain Am J Roentgenol 2001 15 1205 1210 10.2214/ajr.177.5.1771205 11641204

[B40] Veldhuijzen DS Nemenov MI Keaser M Zhuo J Gullapalli RP Greenspan JD Differential brain activation associated with laser-evoked burning and pricking pain: An event-related fMRI study Pain 2009 15 104 113 10.1016/j.pain.2008.10.027 19058914 PMC6449044

[B41] Stern J Jeanmonod D Sarnthein J Persistent EEG overactivation in the cortical pain matrix of neurogenic pain patients Neuroimage 2006 15 721 731 10.1016/j.neuroimage.2005.12.042 16527493

[B42] Absinta M Rocca MA Colombo B Falini A Comi G Filippi M Selective decreased grey matter volume of the pain-matrix network in cluster headache Cephalalgia 2012 15 109 115 10.1177/0333102411431334 22174349

[B43] Peelen MV Downing PE Within-subject reproducibility of category-specific visual activation with functional MRI Hum Brain Mapp 2005 15 402 408 10.1002/hbm.20116 15852382 PMC6871698

[B44] Buvanendran A Ali A Stoub TR Berger RA Kroin JS The use of brain positron emission tomography to identify sites of postoperative pain processing with and without epidural analgesia Anesth Analg 2007 15 1784 1786 10.1213/01.ane.0000270206.30333.cb 18042883

[B45] Minassian AT Ricalens E Humbert S Duc F Aube C Beydon L Dissociating Anticipation from perception: Acute pain activates default mode network Hum Brain Mapp 2013 15 9 2228 2243 10.1002/hbm.22062 22438291 PMC6870109

[B46] Rocca MA Messina R Colombo B Falini A Comi G Filippi M Structural brain MRI abnormalities in pediatric patients with migraine J Neurol 2014 15 2 350 357 10.1007/s00415-013-7201-y 24305994

[B47] Basselin M Villacreses NE Chen M Bell JM Rapoport SI Chronic carbamazepine administration reduces N-Methyl-D-Aspartate receptor–initiated signaling via arachidonic acid in rat brain Biol Psychiatry 2007 15 8 934 943 10.1016/j.biopsych.2007.04.021 17628508 PMC2131715

[B48] Rodriguez-Raecke R Niemeier A Ihle K Ruether W May A Brain gray matter decrease in chronic pain is the consequence and not the cause of pain J Neurosci 2009 15 13746 13750 10.1523/JNEUROSCI.3687-09.2009 19889986 PMC6666725

